# From eyes’ microtremors to critical flicker fusion

**DOI:** 10.1371/journal.pone.0325391

**Published:** 2025-06-09

**Authors:** Pedro Lencastre, Rujeena Mathema, Pedro G. Lind

**Affiliations:** 1 Department of Computer Science, OsloMet - Oslo Metropolitan University, Oslo, Norway; 2 OsloMet Artificial Intelligence Lab, OsloMet, Oslo, Norway; 3 Kristiania University of Applied Sciences, Oslo, Norway; 4 Simula Research Laboratory, Numerical Analysis and Scientific Computing, Oslo, Norway; Shanghai Jiao Tong University, CHINA

## Abstract

The critical flicker fusion threshold (CFFT) is the frequency at which a flickering light source becomes indistinguishable from continuous light. The CFFT is an important biomarker of health conditions, such as Alzheimer’s disease and epilepsy, and is affected by factors as diverse as fatigue, drug consumption, and oxygen pressure, which make CFFT individual- and context-specific. Other causal factors beyond such biophysical processes are still to be uncovered. We investigate the connection between CFFT and specific eye-movements, called microtremors, which are small oscillatory gaze movements during fixation periods. We present evidence that individual differences in CFFT can be accounted by microtremors, and design an experiment, using a high-frequency monitor and recording the participant’s eye-movements with an eye-tracker device, which enables to measure the range of frequencies of a specific individual’s CFFT. Additionally, we introduce a classifier that can predict if the CFFT of specific participant lies in the range of high or low frequencies, based on the corresponding range of frequencies of eyes’ microtremors. Our results show an accuracy of 85% for a frequency threshold of 60 Hz and 88% for a threshold of 120 Hz.

## Introduction

The critical flicker fusion threshold (CFFT) is defined as the frequency at which a periodic light stimulus transitions from appearing flickering to appearing continuous to an observer, thereby serving as an indicator of the temporal resolution capacity of the visual system. The CFFT is assumed to be limited by the temporal resolution of the human photoreceptors in the central retina [1]. This quantity has inter- and intra-individual variation [2] and is reduced by factors such as increased age [3], brain injuries [4], fatigue [5], or alcohol and marijuana [6] consumption. In contrast, a higher CFFT indicates higher levels of concentration and cognitive performance [7] and can be affected by factors such as oxygen pressure [8] or ambient luminance [1]. The CFFT can also be diminished by various medical conditions such as multiple sclerosis, epilepsy [9], Alzheimer’s disease [10], dyslexia, autism [11], hepatic encephalopathy [12] and eye diseases such as cataract [13], optic neuritis and ischemic neuropathy or demyelinating optic neuritis [14].

While the temporal resolution of the visual system can be investigated by recording its electrophysiological response using an electroretinogram [15], it is more commonly assessed through psychophysical methods that measure the individual’s CFFT value. There are several psychophysical paradigms for measuring CFFT, and the results can vary depending on the specific paradigm used. However, despite these variations, a strong correlation has been observed between CFFT values obtained from different protocols, with Pearson coefficients ranging from 0.6 to 0.92 [1]. Given the heterogeneity of experiments and equipment used, reports of CFFT in humans show considerable variation, with values in ranges from 20 to 85 Hz, depending on the specific study conducted [2,10,16–18]. This interval of frequencies is consistent with the temporal resolution of human photoreceptors in the central retina, which is usually limited to CFFTs around 50 Hz [19,20].

Many environmental and health conditions that affect CFFT also influence eye-gaze movements, which can be tracked using eye-tracking technology. Examples of such health conditions include neurodevelopmental conditions such as autism [21] or ADHD [22], dyslexia, drug consumption [23], emotional states, fatigue [24] and level alertness and concentration [25]. Despite some research connecting both topics, eye-gaze dynamics and CFFT, investigations into how they relate to each other are still lacking.

Generally, eye-movements can be classified into two distinct alternating subprocesses: saccades and fixations [26]. The first type of eye-movement, saccades, are rapid jerk-like movements of the eyes that shift the focus of gaze from one point to another. They serve to position the eyes so that the next fixation can occur on a new point of interest. During a fixation, visual information is gathered and processed by the brain. This stationary period is essential for detailed visual perception and cognitive processes such as reading, object recognition, and scene analysis. During everyday activities, our eyes constantly alternate between these saccades and fixations enabling us to gather detailed information about specific aspects of our environment while also scanning for new information.

Our study of eye-movements is focused mainly on fixations, which can be divided into three main subtypes. The first is the so-called microsaccades, a smaller version of saccades that occur involuntarily when an individual is fixating on an area for prolonged periods of time or voluntarily when an individual is aiming to do a small gaze relocation - during reading, for example. It is speculated that microsaccades are important to prevent the retinal image from fading [27].

The second fixational movement is ocular drift, a type of movement that resembles a random walk as it changes direction frequently, in a seemingly erratic manner, resulting in relatively small amplitude displacements of the gaze position [28]. It has recently been shown that this apparently random motion is actually quite reactive to external stimuli [29].

Finally, and more relevant to our study, we have microtremors, an oscillatory movement between 70 Hz to 150 Hz which is smaller than the other fixational movements [30,31]. These movements are difficult to measure and consequently are not studied often [32]. It is hypothesized that such microtremors are caused by asynchronous neuron firing [33], but it is also possible that, since vision is not uniform within the foveola, this targeted microscopic eye-movements may compensate for this lack of homogeneity [34].

This paper is organized as follows: in the next section, we describe our experimental setup to measure CFFT and eye-tracking simultaneously. We also detail the methods used to characterize the periodic behavior of eye-movements. In the results section, we analyze the distribution of CFFT among different participants and address the relationship between microtremor frequency and CFFT variations. As we will see, our findings suggest that the periodicity of eye-gaze shifts correlates significantly with CFFTs. Finally, in the “discussion and conclusions" section, we close the paper with an overview of the main findings, addressing the limitations of our study and stating possible new targets for future research.

## Materials and methods

### Experimental design and setup

**Fig 1:**
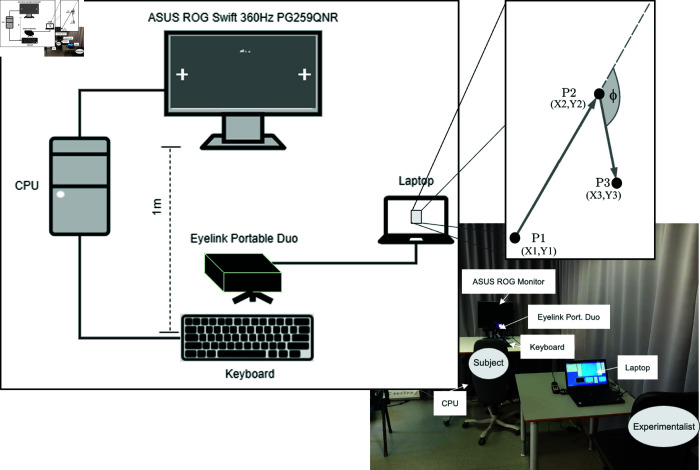
Sketch of the experimental setup: on the high-frequency screen (ASUS ROG Swift 360 Hz PG259QNR) one sees the screenshot shown to each participant, namely two targets, one of them flickering with frequency f. In the inset, we illustrate some of the variables recorded during each session, namely gaze positions (*X* and *Y*) and the angle ϕ between consecutive gaze shifts, as indicated by points P1, P2, and P3. (cf. Eqs (1) and (2)).

We collected data for this study in the eye-tracker lab of Oslo Metropolitan University using a high-frequency monitor (ASUS ROG Swift 360 Hz) [35] and an eye-tracker (Eyelink Portable Duo [36]). The monitor has 1920x1080 pixels and participants used a chin stabilizer and were seated at a 1-meter distance from the screen. Fig 1 (left plot) illustrates the experimental setup, with a screenshot of the performed task in each session (left plot). The right plot illustrates three consecutive eye-gaze positions, highlighting the different variables that will be used for the data analysis (see below).

Binocular eye-tracker data were collected from 85 adult participants, primarily recruited on campus. Among these, 4 participants were excluded given the high number of blinks, leaving a total of 81 participants with a mean age of 23.4 years old, 49 of which are male. While the participant pool includes a significant number of individuals with high education levels, no other threats to validity were identified. The dataset contains records of gaze positions for each eye separately; however, we chose to analyze only the right eye, consistent with common practice in the literature. Data collection took place from 01.Jan.2023 to 01.Dec.2024 and written consent was provided, according to the application Ref. 129768 approved by the Norwegian Ethics Commission (SIKT). The anonymized data, as well as the code for the analysis, has been made publicly available [37].

In the task presented to participants, two stimulus crosses, one left and one right, were shown on the monitor. The crosses were white in color and were placed on a uniform gray background. The gray color was obtained by mixing equal parts of black and white. The crosses had 2.5 cm in width and height, corresponding to $1.4^{\circ }$ of visual angle and were 35 cm apart from each other (corresponding to $19^{\circ }$ of visual angle). The luminance level of the room was 88 lux and the monitor’s brightness was measured at 250 nits. Before the experiment began, participants were informed that one of the two crosses would flicker and their task was to identify the flickering cross at the end of each trial. No gaze direction instruction was given and the participants were free to look at each cross individually. In the task, one of the crosses was flickering at frequencies f=30,60 and 120 Hz, while the other was continuously shown on the screen with opacity 0.125,0.25, and 0.5, increasing with flickering frequency.

The experiment consisted of 9 trials in total, 3 for each of the different values of *f*, and the position of the flickering cross was randomized across trials. Participants were then asked to identify which of the displayed crosses was flickering (left or right). They were also given the choice of answering “Not sure". If participants could correctly identify the flickering cross 2 out of 3 times, then we assume that they could identify flickers. Considering a binomial distribution with 1/3 for the probability of a successful random choice and with the requirement that at least 2 successful choices must happen, the total probability is 7/27~1/4. So, the number of incorrect answers was negligible if less than 25% is observed.

### Eye-gaze classifiers and data processing

#### Eye-gaze velocity and angle between consecutive relocations.

While there are many algorithms to distinguish fixations and saccades, here we will use the native eye-tracker algorithm from *SR Research Ltd.* [38]. This algorithm is a widely accepted standard in the field and was employed due to its demonstrated reliability and accuracy in determining fixations and saccades from eye-tracking data [39]. In this study we will be focused mainly on gaze velocities. Given a series of positions on a plane with coordinates $\left(X_{t},Y_{t}\right)$ we define:

$$⃗v(t)=\left(v_{x}(t),v_{y}(t)\right),$$ (1a)

$$v_{x}(t)=\frac{X_{t+\tau }-X_{t}}{\tau },$$ (1b)

$$v_{y}(t)=\frac{Y_{t+\tau }-Y_{t}}{\tau },$$ (1c)

where τ represents the sampling time of recording equipment (1 ms).

To study the period of micro-tremors, we introduce the angle:

$$\phi (t,t^{\prime })=\arccos\left(\frac{⃗v(t)\cdot ⃗v(t^{\prime })}{\left\|⃗v(t)\|\|⃗v(t^{\prime })\right\|}\right),\;\phi \in [0,\pi ]\;,$$ (2)

for two distinct instants, *t* and $t^{\prime }$. Fig 1 shows the eye-gaze positions $\left(X_{t},Y_{t}\right)$ for three illustrative measurements, together with the corresponding angle ϕ.

Four other variables related to gaze velocity will be important to study the periodic nature of eye-movements. These are:

$$v_{x,\text{unit}}(t)=\frac{v_{x}(t)}{\sqrt{v_{x}^{2}(t)+v_{y}^{2}(t)}},$$ (3a)

$$v_{y,\text{unit}}(t)=\frac{v_{y}(t)}{\sqrt{v_{x}^{2}(t)+v_{y}^{2}(t)}}.$$ (3b)

$$v_{x,\text{fix}}(t)=\frac{X_{t+\tau ,\text{fix}}-X_{t,\text{fix}}}{\tau },$$ (3c)

$$v_{y,\text{fix}}(t)=\frac{Y_{t+\tau ,\text{fix}}-Y_{t,\text{fix}}}{\tau },$$ (3d)

Here, $v_{x,\text{unit}}$ and $v_{y,\text{unit}}$ are the horizontal and vertical components of the normalized gaze velocity. The components $v_{x,\text{fix}}$ and $v_{y,\text{fix}}$ are the horizontal and vertical fixational gaze velocities, which are calculated in the same way as the gaze velocity but only considering the data points where both locations $\left(X_{t},Y_{t}\right)$ and $\left(X_{t+\tau },Y_{t+\tau }\right)$ are labeled as fixations (‘fix’). Fixational gaze velocities are relevant to this study since microtremors are subtle movements that could easily be obfuscated by faster dynamics such as the ones responsible for larger gaze shifts.

#### Power spectrum and fast fourier transform.

When studying periodic movements, one of the most important tools is the Fourier transform, a mathematical operation that translates a signal from its time domain into the frequency domain and is foundational for analyzing the frequency components of signal. Here, we compute it by using the Fast Fourier Transform (FFT), an algorithm which enables the efficient computation of the Discrete Fourier transform (DFT). The DFT, specifically, deals with discrete signals, representing them as a sum of sinusoidal functions, each with a specific frequency, amplitude, and phase. However, directly computing the DFT is computationally intensive, requiring *O*(*N*^2^) operations for *N* data points. This complexity makes the DFT impractical for large datasets, necessitating a more efficient approach.

The FFT addresses this challenge by dramatically reducing the computational burden to O(NlogN). This efficiency is achieved through a strategy, which recursively breaks down the DFT of a sequence of values into smaller DFTs. By exploiting symmetries in the computation, specifically the periodicity and parity properties of the sine and cosine functions, the FFT minimizes redundant calculations. We follow the Cooley-Tukey implementation, which recursively splits a DFT of any composite size $N=N_{1}\cdot N_{2}$ into many smaller DFTs, combining their results to produce the final transform.

In this study, we use the Fourier transform to calculate the power spectrum, which shows how the power or variance of the signal is distributed across different frequencies. As the name indicates, the power spectrum gives an indication of the power associated with each frequency. The power spectrum Ps(k) is calculated from the Fourier transform F(k) by squaring the magnitude of the Fourier transform, i.e., *Ps*(*k*) = |*F*(*k*)|^2^. We will compute the power spectrum for the horizontal and vertical components of the fixation velocity ${\vec{v}}_{\text{fix}}(t)=(v_{x,\text{fix}}(t),v_{y,\text{fix}}(t))$ and the unitary velocity ${\vec{v}}_{\text{unit}}(t)=(v_{x,\text{unit}}(t),v_{y,\text{unit}}(t))$.

#### Empirical mode decomposition.

Another method which we will use to study periodic movements is the empirical mode decomposition (EMD), an adaptive and data-driven method for decomposing a complex signal into a set of simpler intrinsic mode functions (IMFs). The EMD process begins by identifying the local extrema (both maxima and minima) of the signal. These extrema are then used to create upper and lower envelopes through spline interpolation. The mean of these envelopes is subtracted from the original signal to produce a first approximation of an IMF. This process, known as sifting, is repeated iteratively on the residual (the original signal minus the extracted IMF) until the resulting function meets the criteria for an IMF: it must have the same number of zero crossings and extrema, and its envelopes, as defined by the local maxima and minima, must be symmetric around zero. Once an IMF is extracted, the sifting process continues on the residual signal until all significant IMFs are obtained, leaving a final residual that represents the trend of the original signal. The resulting IMFs represent the signal’s intrinsic oscillatory modes, ordered from high to low frequency.

In this context, three important quantities to analyze IMFs can be computed. The first two quantities are the instantaneous phase (IP) and the instantaneous amplitude (IA), which are obtained with the help of the Hilbert-Huang [40] transform to the IMFs. Mathematically, if *S*(*t*) represents the IMF of the original signal, the Hilbert-Huang transform H{S(t)} and the respective IMF, yield a signal *Z*(*t*) decomposable into the IA *a*(*t*) and the IP θ(t), namely

$$Z(t)=S(t)+jH\{S(t)\}=a(t)e^{j\theta (t)}\;,$$ (4)

where *j* is the imaginary unit (*j*^2^ = −1). The IP θ(t) can, therefore, be expressed as

θ(t)=arg(S(t)+jH{S(t)}), (5)

while the IA is the magnitude of the analytic signal, namely a(t)=|Z(t)|.

The third quantity is the instantaneous frequency (IF), which is derived from the IP by taking its temporal derivative, offering a dynamic representation of the frequency content of the signal as it evolves over time:

$$\omega (t)=\frac{d\theta (t)}{dt}\;.$$ (6)

These three quantities allow for a comprehensive analysis of non-stationary and nonlinear signals, capturing the time-varying amplitude and frequency characteristics that traditional Fourier-based methods might miss.

### Decision trees as classifiers of CFFT ranges

Decision trees are a powerful and intuitive machine learning technique that employs a flowchart-like structure for both classification and regression tasks. At the core of this technique is a tree-like model where the root node represents the initial decision point based on a feature of the data. From this root, subsequent branches emanate, each corresponding to a possible outcome of the initial decision. These branches continue to split based on additional features, forming a hierarchical structure that ultimately leads to leaf nodes. Each leaf node represents a final classification or prediction, making the decision tree a comprehensive model that encapsulates a series of decisions leading to a particular outcome.

One of the key strengths of decision trees is their clarity and interpretability. The hierarchical structure can be easily visualized, providing a transparent view of the decision-making process. This transparency is particularly valuable for understanding how different features contribute to the final outcome, which is beneficial for both model evaluation and stakeholder communication. By mapping out the relationships between features and their corresponding outcomes, decision trees enable a straightforward analysis of the underlying patterns in the data. This makes them not only useful for predictive analytics but also for gaining insights into the data itself. Despite their simplicity, decision trees can be highly effective, especially when combined with techniques like pruning to avoid overfitting or integrated into ensemble methods such as random forests to enhance predictive performance. In this study, this classification method is going to be used to determine if a particular individual has a CFFT above 60 Hz or 120 Hz based on the characteristics of the gaze fixation’s periodic behaviour. The ground truth to assess the performance of tree-based classifiers is given by the direct answer of each participant, either reporting to have/ have not seen the flicker.

## Results

### Measuring CFFT and the periodicity of eye-gaze microtremors

**Fig 2:**
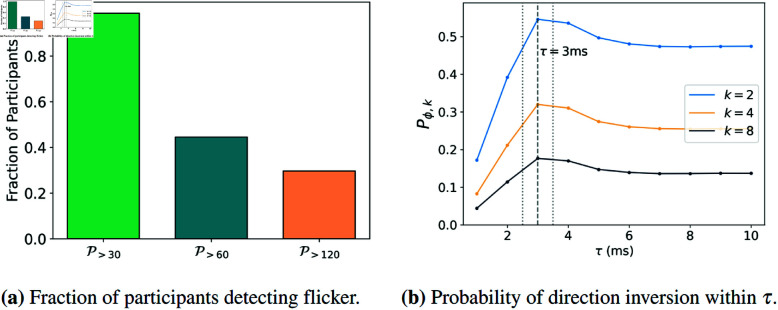
(a) Fraction of participants $P_{>f}$ who were able to detect the flicker at a frequency of f=30,60 and 120 Hz (total 81 participants). $P_{<f}=1-P_{>f}$, indicates the participants who did not detect flicker at these frequencies. (b) Probability ${\text{Pr}}_{\phi ,k}(\tau )$ of the angle ϕ(t,t+τ) being within the range $\left[\frac{k-1}{k}\pi ,\pi \right]$ in a time interval [t,t+τ], i.e. the probability for the gaze to invert its direction within that time interval (cf. Eq (2) and Fig 1, right plot). We observe an increasing probability ${\text{Pr}}_{\phi ,k}(\tau )$ for small τ with a maximum at τ≃3±0.5 ms (dashed vertical line), indicating that gaze movements revert direction every 2.5 ms to 3.5 ms approximately. Here, the parameter *k* determines how narrow the angle range must be for a movement to be classified as a reversal in direction. The reversion corresponds to a period between 5 and 7 ms, i.e. a frequency between 142 Hz and 200 Hz. These values are consistent with the larger microtremor frequency values reported in the literature [30] (see text).

We observed that 80 out of 81 participants could see the cross flickering at 30 Hz. In particular, we verified that only 3 out of 792 trials had an incorrect answer of whether the target was flickering or not. As shown in Fig 2(a), we counted the number of participants who could distinguish flickering at each one of the three frequencies, f=30,60 and 120 Hz. The left plot shows the fraction $P_{>f}$ of those participants for each frequency *f*. In each case, $P_{<f}=1-P_{>f}$.

At a frequency of 60 Hz, around half the participants were able to identify the flickering, while at 120 Hz, the fraction reduced to ~30%. A flicker frequency of 120 Hz is higher than the thresholds reported in the literature. However, we show evidence that a significant fraction of participants still identifies flickering at such high frequency. Moreover, the plot in Fig 2(a) enables to estimate a sort of typical length of frequency interval. This length corresponds to a decay of 1/*e* of the number of individuals able to identify flickering within the full range of frequencies. We discuss this further below in the discussion section.

To study the periodicity of eye “tremors", we considered the probability Pr for the angle ϕ(t,t+τ) to take values around π. Values of ϕ(t,t+τ) close to π represent a reversal in gaze direction, which is qualitatively what tremors are. This probability is estimated as the fraction of time-steps when the angle ϕ is in the range [π − ϵ,π  +  ϵ] for some (small) ϵ>0. Since ϕ represents an angle between vectors, i.e. it takes values in [0,π], we computed the fraction of time-steps in intervals [π − ϵ,π], with $\epsilon =\frac{1}{k}$. Specifically, we computed the probability for ϕ(t,t+τ) to take values in the range $A_{k}=[(1-\frac{1}{k})\pi ,\pi ]$, for different values of *k*. Note that the interval *A*_*k*_ has a length of 1/*k* which decreases with *k*. In Fig 2(b) plotted the result of ${\text{Pr}}_{\phi ,k}$ for k=2,4 and 8 as a function of τ (see Eq (1)). We observed that, for τ∈[1,2] (in ms), the gaze movements typically fall outside the interval of ϕ(t,t+τ)-values, indicating to keep the moving direction within the interval [t,t+τ]. As τ increases, the probability for ϕ(t,t+τ) to lie within the interval *A*_*k*_ increases as well, with a maximum at τ∈[3,4] ms. This indicates that the periodicity associated with eye-movements inverting their direction lies between 6 and 8 ms, corresponding to a frequency between 125 Hz and 166 Hz.

### Identifying individuals with high CFFT

After collecting data from 81 participants and assessing their ability to identify flickering at f=30,60 and 120 Hz, we found that only one participant failed to identify the flickering at 30 Hz. Therefore, we will focus our analysis solely on the frequencies of 60 and 120 Hz.

A function that can be used to differentiate between participants who can identify the flickering and those who cannot is the cumulative power spectrum of a certain variable X:

$$C_{X}(f^{\prime })=\int_{0}^{f^{˜}}S_{X}(f˜)df˜\;,$$ (7)

where $S_{X}(f˜)$ is the corresponding normalized power-spectrum. Henceforth, the variable X will be substituted by one of the variables defined in Eq (3). Here, we use $f^{\prime }$ to distinguish the frequency of the power spectrum from the flickering frequency *f* tuned in the experimental task (see above). Note that, since we compute the power spectra using FFT, the normalization is performed by computing the cumulative power over the full spectrum, with a maximum frequency of 500 Hz, i.e. half of the sampling frequency.

**Fig 3:**
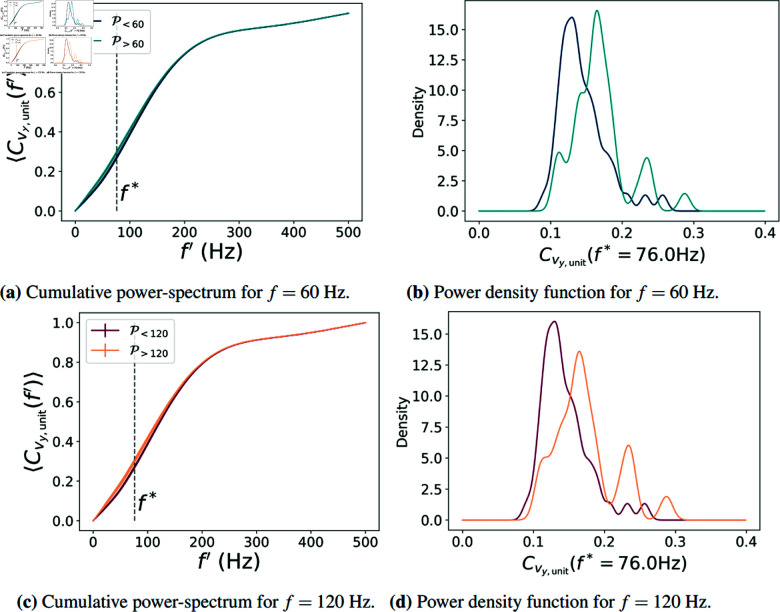
(a) The cumulative power-spectrum $C_{y,\text{unit}}(f^{\prime })$ is shown for groups $P_{<60}$ and $P_{>60}$, together with (b) the probability density function of the values $C_{y,\text{unit}}(f^{*}=76)$, across all 81 participants. In (c) and (d), we have the same plots but for $P_{<120}$ and $P_{>120}$. We used the Mann-Whitney U-test to determine that, for the variable $v_{y,\text{unit}}$, $f^{*}=76$ Hz is the value that best separates the groups $P_{<60}$ and $P_{>60}$ as well as $P_{<120}$ and $P_{>120}$. See also Table 1.

Fig 3(a) and 3(c) shows the cumulative power spectrum, $C_{v_{y,\text{unit}}}(f^{\prime })$, computed as described above for *f* = 60 Hz and *f* = 120 Hz respectively. As expected, the difference in $C_{v_{y,\text{unit}}}(f^{\prime })$ between the $P_{<f}$ and $P_{>f}$ groups varies with $f^{\prime }$. Therefore, it is useful to identify the frequency at which these groups exhibit the most distinct $C_{v_{y,\text{unit}}}(f^{\prime })$ profiles. We henceforth denote by f^*^ the value of $f^{\prime }$ that produces the largest mean difference between groups for a particular variable. In the case of $C_{v_{y,\text{unit}}}$ and the groups $P_{>60}$ and $P_{>120}$ we find an optimal frequency f^*^ = 76 Hz. Fig 3(b) and 3(f) display the distribution of $C_{v_{y,\text{unit}}}(f^{\prime })$ at this frequency, where we observe that the $P_{>f}$ group has a higher mean value. Table 1 lists the frequency f^*^ at which the largest mean differences occur for each variable as well as the corresponding *p*-value from the MWU-test.

**Table 1:** Summary of the variables used to distinguish between the target groups, i.e. between $P_{<60}$ and $P_{>60}$ and $P_{<120}$ and $P_{>120}$ (see text). Using the Fourier spectra of the different velocity components (cf. Eq (7)), the component $v_{y,\text{unit}}$ shows the highest capability to identify participants distinguishing CFFT of 60 Hz, while $v_{y,\text{fix}}$ is best to identify participants distinguishing CFFT of 120 Hz.

Variable	*f* = 60 Hz		*f* = 120 Hz	
	f^*^ (Hz)	*p*-value	f^*^ (Hz)	*p*-value
$C_{v_{x,\text{unit}}}$	52.0	$4.8\times 10^{-2}$	56.0	$1.11\times 10^{-1}$
$C_{v_{y,\text{unit}}}$	76.0	$2\times 10^{-3}$	76.0	$5\times 10^{-3}$
$C_{v_{x,\text{fix}}}$	29.5	$7.43\times 10^{-1}$	29.5	$8.35\times 10^{-1}$
$C_{v_{y,\text{fix}}}$	224.0	$6.52\times 10^{-1}$	224.0	$3.48\times 10^{-1}$
${\text{cdf}}_{v_{x,\text{unit}},S_{1}}$	235.5	$6.04\times 10^{-1}$	146.5	$3.41\times 10^{-1}$
${\text{cdf}}_{v_{x,\text{unit}},S_{2}}$	108.0	$5.65\times 10^{-1}$	107.0	$1.84\times 10^{-1}$
${\text{cdf}}_{v_{x,\text{unit}},S_{3}}$	52.5	$4.11\times 10^{-1}$	46.5	$3.88\times 10^{-1}$
${\text{cdf}}_{v_{y,\text{unit}},S_{1}}$	253.5	$3.0\times 10^{-2}$	207.5	$5.3\times 10^{-2}$
${\text{cdf}}_{v_{y,\text{unit}},S_{2}}$	106.5	$5.27\times 10^{-1}$	106.0	$3.35\times 10^{-1}$
${\text{cdf}}_{v_{y,\text{unit}},S_{3}}$	54.0	$4.33\times 10^{-1}$	54.0	$2.59\times 10^{-1}$
${\text{cdf}}_{v_{x,\text{fix}},S_{1}}$	170.0	$5.52\times 10^{-1}$	190.0	$4.31\times 10^{-1}$
${\text{cdf}}_{v_{x,\text{fix}},S_{2}}$	92.5	$6.93\times 10^{-1}$	96.0	$5.66\times 10^{-1}$
${\text{cdf}}_{v_{x,\text{fix}},S_{3}}$	51.0	$3.95\times 10^{-1}$	52.0	$2.99\times 10^{-1}$
** ${\text{cdf}}_{v_{y,\text{fix}},S_{1}}$ **	** 195.5 **	** $2.5\times 10^{-2}$ **	** 197.0 **	** $3.1\times 10^{-2}$ **
${\text{cdf}}_{v_{y,\text{fix}},S_{2}}$	99.0	$1.07\times 10^{-1}$	100.0	$1.23\times 10^{-1}$
${\text{cdf}}_{v_{y,\text{fix}},S_{3}}$	52.0	$1.73\times 10^{-1}$	53.0	$3.23\times 10^{-1}$

Table 1 (first four rows) shows the values of f^*^ in the cumulative power spectrum, for each velocity component in Eq (3) with the corresponding *p*-value of the MWU-test This test evaluates the null hypothesis that the samples originate from the same distribution; when the distributions are similarly shaped, this is equivalent to testing for equal medians. For *f* = 60 Hz the component $v_{y,\text{unit}}$ shows the highest capability to distinguish between participants who can resolve that CFFT from those who cannot. For a CFFT *f* = 120 Hz the component $v_{y,\text{fix}}$ is the best variable. While there are no significant differences in the *p*-values, it is surprising that the *y*-components of the velocities are the ones with highest confidence, since the *y*-component is typically the noisiest one of the eye-gaze velocity.

When it comes to the decomposition into different IMFs by using EMD, the statistical analysis of the IFs retrieves better results than the power-spectrum analysis above. Similar to the cumulative power spectrum, here we consider the cumulative density function using the IF’s probability density function $\rho_{X,Y}(f˜)$ of each IMFs, namely

$${cdf}_{X,Y}(f)=\int_{0}^{f}\rho_{X,Y}(f˜)df˜\;,$$ (8)

where $\rho_{X,Y}(f˜)$ is the probability density function of the IF, Y refers to a specific IMF, and, as before, X refers to a fixation velocity variable defined in Eq (3). Given the already large number of variables in our analysis, we do not consider both $v_{x,\text{unit}}$ and $v_{y,\text{unit}}$.

**Fig 4:**
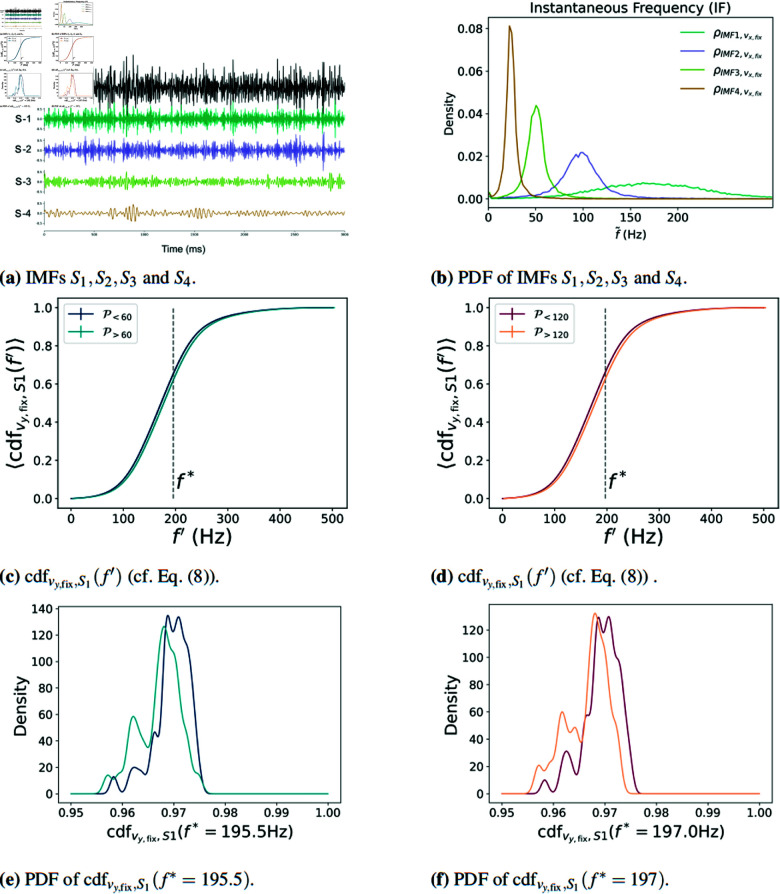
On (a), we illustrate, for a randomly selected participant, a set of four IMFs, $S_{1},S_{2},S_{3}$ and *S*_4_, generated from the EMD of $v_{y,\text{fix}}$, and, on (b), we illustrate with the probability density function of the IF of each IMF for the same participant. Representation of ${cdf}_{v_{y,\text{fix}},S_{1}}(f^{\prime })$ (cf. Eq (13)) for ($P_{<60}$ and $P_{>60}$) (c) and ($P_{<120}$ and $P_{>120}$) (d) respectively. Probability density function of the values of ${cdf}_{v_{y,\text{fix}},S_{1}}(f^{*}=195.5)$ for ($P_{<60}$ and $P_{>60}$) (e) and of ${cdf}_{v_{y,\text{fix}},S_{1}}(f^{*}=197)$ for ($P_{<120}$ and $P_{>120}$) (f) respectively. See also Table 1 for the p-values of the optimal frequency f^*^ and the optimal values of MWU-test.

Fig 4(b) shows an example of the EMD of $v_{y,\text{fix}}$, including four IMFs $S_{1},S_{2},S_{3},S_{4}$ for one randomly selected participant. Fig 44(b) depicts the corresponding probability density function of the IF $\rho_{v_{y,\text{fix}},Y}(f˜)$ is shown with $Y=\{S_{1},S_{2},S_{3},S_{4}\}$. In the Fig 4(c) and 4(e), ${cdf}_{v_{y,\text{fix}},S_{1}}(f^{\prime })$ is represented for $P_{<60}$ and $P_{>60}$ and $P_{<120}$ and $P_{>120}$ respectively, we observe that the values of ${cdf}_{v_{y,\text{fix}},S_{1}}$ are larger for the participants who can detect fast flickering stimuli ($P_{>}$). As before, we use the MWU-test to determine the values of f^*^, i.e. the frequency which maximizes the difference in the means between the $P_{>}$ and $P_{<}$ groups for a particular quantity. The exact values of f^*^ and their corresponding *p*-values are shown in Table 1.

In Table 1 we computed Eq (13) for the horizontal and vertical components of the fixation and unitary velocities. Similarly as for the cumulative power spectrum, each of these quantities is evaluated at the frequency f^*^, which is the one that maximizes the difference between the mean of each quantity in Table 1 for the $P_{>}$ and $P_{<}$ groups.

### Training a classifier and evaluation

In order to check how much the variables in Table 1 determine the CFFT, we create a set of decision tree classifiers, using all possible combinations of the four velocity components. Using a validation set, we employ a five-fold cross-validation for variable selection. We also use cross-validation to tune the hyper-parameters of our classifier, including maximum depth and features, as well as the minimum number of samples required to split an internal nod and the minimum number of samples required to be at a leaf node. Given that the number of participants is not evenly distributed among the groups, we use the synthetic minority oversampling technique (SMOTE) [41] to train the classifier. To evaluate the accuracy of our procedure, we use previously unseen test data, again using a five-fold cross-validation. The classification results are summarized in Table 2, where a detailed breakdown of the true and false positive and negative classifications is presented. We see that the group differences are larger at the 120 Hz threshold (88% accuracy) when compared to the 60 Hz threshold (85% accuracy). Also, either in the case of *f* = 60 Hz or *f* = 120 Hz, we observe higher values of specificity when compared to sensitivity, which indicates that the classifier is better at correctly identifying negatives than positives. The fact that precision is higher than sensitivity suggests that while the classifier reliably labels positive cases as such, its conservative threshold for positive classification likely leads to true positive cases being overlooked.

**Table 2:** Results relative to a set of tree classifiers, exploring all combinations of the four velocity components.

	*f* = 60 Hz	*f* = 120 Hz
True Positives	30	21
False Positives	5	3
True Negatives	39	50
False Negatives	7	7
Accuracy	0.85	0.88
Sensitivity	0.81	0.75
Specificity	0.89	0.94
Precision	0.86	0.86

## Discussion and conclusions

### Managerial implications

As expected from previous studies, we found individual variations on CFFT. Based on our experiment, we thus create 4 non-mutually-exclusive groups, namely the group of participants that could distinguish flickering at 120 Hz (resp. 60), which we label as $P_{>120}$ (resp. $P_{>60}$) and those that could not identify the flickering at 120 Hz (resp. 60), which we designate as $P_{<120}$ (resp. $P_{<60}$). These CFFT values are higher than those reported in the literature. This discrepancy may be attributed to the utilization of a different protocol for measuring CFFT, which can significantly influence the obtained values [1].

From Fig 2, although one has only three points, we can derive a rough estimate of the fraction of participants as an exponential of the frequency, $\exp\left(-f/f_{\ell }\right)$, where $f_{\ell }$ is a measure of the frequency tolerance within which the eyes start losing the ability to resolve flickering images. Such exponential fit yields approximately $f_{\ell }\sim 70$ Hz.

On the other hand, the microtremor range of frequencies is consistent with the higher values reported in previous literature [30], falling within the interval of [70,150] Hz. The uniqueness of our study lies on two major outcomes. First, we provide quantitative evidence on the existence of a link between eye-movements (microtremors) and CFFT. Second, we show that the frequency of periodic movements of the eyes has a direct impact on the CFFT. Moreover, the importance of microtremors for visual perception is supported by previous studies. For example, it has been argued that these eye-movements help prevent retinal adaptation by continuously refreshing the retinal image, thereby maintaining photoreceptor responsiveness [42]. Microtremors are also proposed to enhance visual acuity by enabling a dynamic sampling of the visual scene, which is especially important given that the distribution of visual cones in the retina is not continuous but rather exhibits regional variability [43,44].

### Theoretical contributions

In addition to underscoring the relationship between ocular microtremors and visual perception, our findings may also point towards a reevaluation of the assumptions regarding the neural mechanisms underlying flicker fusion thresholds: besides preventing retinal adaptation, our results also indicate that microtremors modulate the temporal resolution of visual processing. This suggests that flicker perception is influenced by both the limitations of photoreceptors and the dynamics of eye movements.

We have shown with statistical significance, according to the MWU-test, that there are differences in the eye’s periodic movements for individuals with different CFFTs. Furthermore, an analysis of eye movements can determine if an individual’s CFFT is above 60 Hz with an 85% accuracy (88% accuracy for the 120 Hz case). Furthermore, using EMD, we observe that the significant differences in the eye’s periodic motion between the $P_{<}$ and $P_{<}$ occur at a frequency close to the range of microtremor frequencies, namely around 200 Hz.

### Future directions

Several potential improvements and future directions can be considered. Future studies may overcome two of the main limitations of our approach, namely measuring CFFT on a continuous range of frequencies and not just above or below 60 and 120 Hz and using special equipment to measure microtremors such as a video-oculography system [45]. Standardizing these protocols can improve the reliability and comparability of results across studies. Conducting longitudinal studies can help understand how CFFT and microtremors change over time and under different conditions, providing insights into their potential as biomarkers for disease progression or treatment efficacy. Ensuring more stringent control over external variables such as lighting, fatigue levels, and cognitive load during experiments can help isolate the specific impact of microtremors on CFFT.

Combining eye-tracking data with neuroimaging techniques (e.g., fMRI, EEG) can lead to the identification of the neural mechanisms underlying the relationship between CFFT and eye movements, providing insights into specific brain regions involved and their roles in health and disease. Furthermore, our classification results could be improved by checking a larger number of simultaneous variables for the decision tree, considering more variables, or using a more complex method such as random forests or gradient boosting [46].

Given the improvements in camera hardware and video-based eye-tracking [47], these results may provide a quick screening for the diseases mentioned above or have a quick assessment of an individual’s fitness to, for example, drive or engage in other concentration-heavy duties. Since we are dealing mainly with the frequency of periodic movements, there may be reduced calibration requirements to measure this movement with video-based eye tracking.

Finally, the subtle nature of microtremors and the consequential difficulty in measuring them have prevented microtremors from playing a very significant role in the medical applications of eye-tracking when compared to the usual analysis of fixations and saccades. Given that CFFT and microtremors are interrelated, it is natural to assume that conditions that affect the first will also impact the second. This points towards a transformative role for this type of eye-movement as early biomarkers of neurodegenerative and neurodevelopmental conditions. Using a similar methodology to ours, the hypothesis that microtremors can be used to detect neurodevelopmental conditions can be tested.
